# The potential of C4 grasses for cellulosic biofuel production

**DOI:** 10.3389/fpls.2013.00107

**Published:** 2013-05-03

**Authors:** Tim van der Weijde, Claire L. Alvim Kamei, Andres F. Torres, Wilfred Vermerris, Oene Dolstra, Richard G. F. Visser, Luisa M. Trindade

**Affiliations:** ^1^Wageningen UR Plant Breeding, Wageningen University and Research CentreWageningen, Netherlands; ^2^Department of Microbiology and Cell Science and Genetics Institute, University of FloridaGainesville, FL, USA

**Keywords:** biofuel, C4 grasses, yield, lignocellulose, biomass quality, plant breeding, miscanthus, maize

## Abstract

With the advent of biorefinery technologies enabling plant biomass to be processed into biofuel, many researchers set out to study and improve candidate biomass crops. Many of these candidates are C4 grasses, characterized by a high productivity and resource use efficiency. In this review the potential of five C4 grasses as lignocellulosic feedstock for biofuel production is discussed. These include three important field crops—maize, sugarcane and sorghum—and two undomesticated perennial energy grasses—miscanthus and switchgrass. Although all these grasses are high yielding, they produce different products. While miscanthus and switchgrass are exploited exclusively for lignocellulosic biomass, maize, sorghum, and sugarcane are dual-purpose crops. It is unlikely that all the prerequisites for the sustainable and economic production of biomass for a global cellulosic biofuel industry will be fulfilled by a single crop. High and stable yields of lignocellulose are required in diverse environments worldwide, to sustain a year-round production of biofuel. A high resource use efficiency is indispensable to allow cultivation with minimal inputs of nutrients and water and the exploitation of marginal soils for biomass production. Finally, the lignocellulose composition of the feedstock should be optimized to allow its efficient conversion into biofuel and other by-products. Breeding for these objectives should encompass diverse crops, to meet the demands of local biorefineries and provide adaptability to different environments. Collectively, these C4 grasses are likely to play a central role in the supply of lignocellulose for the cellulosic ethanol industry. Moreover, as these species are evolutionary closely related, advances in each of these crops will expedite improvements in the other crops. This review aims to provide an overview of their potential, prospects and research needs as lignocellulose feedstocks for the commercial production of biofuel.

## From biomass to biofuel

The growing global consumption of finite fossil fuel resources and the negative climatic consequences thereof are currently driving a search for renewable alternatives that bring the promise of energy security and sustainability (Charles et al., 2007). The successful replacement of oil as industrial raw material will depend largely on biomass processing techniques, as energy from biomass, in contrast to nuclear, wind, water and photovoltaic energy, can be stored as a liquid energy carrier in the form of biofuels (Perlack et al., 2005; Wyman, 2007; Karp and Halford, 2010). As such, it is currently the only alternative amenable to replace fossil fuels to support mobility on large scales (Wyman, 2008).

The production of biofuel from plant carbohydrates depends on the solar energy stored in plant biomass in the form of soluble sugars, starch and structural polysaccharides through photosynthesis. At the moment, the main pathway to convert these carbohydrates into biofuel is through biochemical extraction and fermentation to produce bioethanol (Balat, 2011). Structural polysaccharides constitute the bulk of all plant biomass, since they are the intrinsic components of the plant cell wall, and are by far the most abundant carbohydrates. However, currently most bioethanol is produced from soluble sugars and starch, as they are more easily processed into biofuel than cell wall polysaccharides (Naik et al., 2010). The plant cell wall, in particular the secondary cell wall, is a rigid, protective structure that confers stability and resistance to degradation. This is due to its main constituents—the structural polysaccharides cellulose and hemicellulose and the phenolic polymer lignin—and their interlinking into an unyielding matrix (Himmel et al., 2007; Zhao et al., 2012). Bioethanol production from the cell wall fraction of plant biomass, referred to as lignocellulose, requires a pretreatment to loosen the structure of the cell wall. During this process, combinations of heat, pressure and chemicals are applied to disrupt the crosslinks between the main cell wall constituents and to improve the exposure of the polysaccharides to the enzymatic hydrolysis (Mosier et al., 2005; Zheng et al., 2009). Hydrolysis is required to disassemble cellulose and hemicellulose into their monomeric sugar constituents, which can subsequently be fermented into bioethanol (Balat, 2011). Structural polysaccharides represent the most abundant carbon resource for large-scale biofuel production, with no or very limited use in food and feed applications (Farrell et al., 2006; Wyman, 2007; Balat et al., 2008).

There is extensive interest in cellulosic ethanol, since biomass is considered a low-cost feedstock, which is available in massive quantities and can often be locally produced. A comparative study of gasoline and cellulosic ethanol with respect to net energy and net greenhouse gas emissions showed cellulosic ethanol to have 94% lower greenhouse gas emissions (Schmer et al., 2008). Hence, cellulosic ethanol can contribute to an environmentally sustainable supply of energy and simultaneously bring the promise of energy security (Farrell et al., 2006; Wyman, 2007). This has promoted major private and public investments in several demonstration and pilot scale cellulosic ethanol plants. Although some of these facilities are operational, none of them are producing cellulosic ethanol at a true commercial scale to date. This is evidenced by the fact that the combined cellulosic ethanol production of such facilities in the United States, once estimated to exceed 750 million liters by 2012 (Coyle, 2010), is currently still stuck at a mere 30 million liters per year (RFA, 2012).

The difficulties that impede scaling up cellulosic ethanol production to a commercial level include infrastructural challenges and high capital and operating costs (Richard, 2010). Infrastructural challenges arise from the difficulties associated with the low density of biomass feedstocks. The costs of transport and storage of large volumes of biomass are high compared to those for fossil energy carriers, with a much higher energy density. Depending on the location of the ethanol plant, feedstock costs are estimated to account for ~38% of the plant's operating costs (Gnansounou and Dauriat, 2010). The larger the facility, the more complicated and expensive transportation may become, as hauling distance increases with increasing biomass supply demands. However, the smaller the facility, the longer it takes to get a positive return on investment in capital costs for the setup of a specialized plant, equipped with pretreatment reactors and saccharification and fermentation tanks made of non-corroding materials (Richard, 2010). The major hurdle with respect to the cost competitiveness of lignocellulose conversion technologies is the high input of energy and chemicals required to extract and hydrolyze cell wall carbohydrates (Wyman, 2007). The pretreatment procedure may account for up to 25% of the total processing expenses, due to the stringent processing conditions required to make the cell wall carbohydrates sufficiently accessible to enzymatic hydrolysis (Gnansounou and Dauriat, 2010).

To increase the profitability of biomass conversion platforms it is vital that a low cost lignocellulose feedstock is exploited. Hence, it is envisioned that agricultural, municipal, and forestry biomass residues are the main substrates of the first cellulosic biorefineries, as they represent widely available, low-cost feedstocks. In the United States projections have been made to estimate the amount of biomass supply that will be potentially available by 2030 (Perlack et al., 2005). Of the projected total of 1366 million dry tonnes of biomass, 621 million tonnes are agricultural residues generated from 157 million hectares of arable land. To provide the additional supply, high-yielding biomass crops are envisioned that are optimized for biofuel purposes. Probably, their cultivation will be in part limited to marginal lands, in order to minimize competition with food and feed production. Therefore, optimization of these crops to low input conditions is desirable, as are breeding efforts to improve tolerance to drought and nutrient use efficiency, since irrigation and fertilization are both costly and unfavorable from a sustainability perspective.

In addition, biomass quality is seen as a very important breeding objective. In this context, biomass quality pertains to the amenability of the lignocellulose feedstock for industrial conversion into bioethanol. Realizing the potential of cellulosic ethanol, but also the agronomical and physiological requirements that future bioenergy cropping systems must comply to, many researchers set out to identify, investigate and enhance candidate biomass crops. Effectively, their main objectives are to (1) maximize the supply of lignocellulose in a sustainable and cost-effective way, and (2) improve the conversion efficiency of lignocellulosic biomass into ethanol. The development of dedicated bioenergy crops is envisioned to substantially reduce the production costs of cellulosic ethanol and thus contribute to the establishment of a viable cellulosic ethanol industry.

## C4 grasses as lignocellulosic feedstocks

### The benefits of C4 photosynthesis

One of the most important factors in the selection of energy crops is their high yield potential for biomass production. A high efficiency of CO_2_ fixation into biomass is therefore of chief importance for energy crops, although biomass yield is determined by a number of other factors as well. The efficiency of CO_2_ fixation is primarily determined by the type of photosynthesis found in a plant species.

The predominant form of photosynthesis amongst terrestrial plants is the C3 type of photosynthesis, in which CO_2_ is fixated by ribulose biphosphate carboxylase oxygenase (Rubisco) (Ehleringer and Cerling, 2002). The efficiency of carbon fixation by Rubisco, however, is often compromised, as the enzyme has a dual role and may bind O_2_ instead of CO_2_ as a substrate, especially at higher temperatures and low atmospheric CO_2_ conditions (Sage et al., 2012). This oxygenase reaction eventually leads to the production of CO_2_ in a process known as photorespiration (Ehleringer and Cerling, 2002).

C4 photosynthesis is a morphological and biochemical modification of C3 photosynthesis in which Rubisco oxygenase activity is reduced due to a CO_2_ concentrating mechanism that involves phospho*enol*pyruvate (PEP) carboxylase (Ehleringer and Cerling, 2002).

Due to their photorespiration-suppressing modifications, C4 plants have a higher potential efficiency of converting solar energy to biomass (Ehleringer and Cerling, 2002; Zhu et al., 2008), evidenced by the fact that 11 out of the 12 most productive plant species on Earth are C4 species (Furbank, 1998). In addition, the C4 mechanism is intrinsically linked to 1.3–4 times higher nitrogen use efficiency (NUE) and water use efficiency (WUE) (Sage and Zhu, 2011), owing to a respective reduction in leaf protein content and stomatal conductance (Taylor et al., 2010; Byrt et al., 2011; Ghannoum et al., 2011; Sage and Zhu, 2011). The former arises from a reduction in the amount of photosynthetic proteins required for optimal photosynthesis (Ghannoum et al., 2011). The higher WUE is associated with a faster fixation of CO_2_ by the O_2_-insensitive PEP carboxylase. Therefore, the time stomata are required to be open for the uptake of CO_2_ is shorter, leading to a reduction of leaf water evaporation (Byrt et al., 2011).

C4 photosynthesis is considered an advantageous characteristic for biomass crops, especially considering that most future climate scenarios predict an increase in dry and saline areas and erratic rainfall, conditions in which the advantages of C4 photosynthesis over the C3 type are even more apparent (Byrt et al., 2011). However, in colder regions of the world C3 plants may outperform C4 species as bioenergy crops (Carroll and Somerville, 2009).

For the sake of being complete, a third type of photosynthesis exists, the crassulacean acid metabolism (CAM), which is employed by cactuses and succulents. These species are not deemed primary candidates for biomass production (Vermerris, 2008), although they may be productive in some extreme environments unsuitable for other species (Youngs and Somerville, 2012).

### Promising C4 grasses for the industry

**Figure 1:**
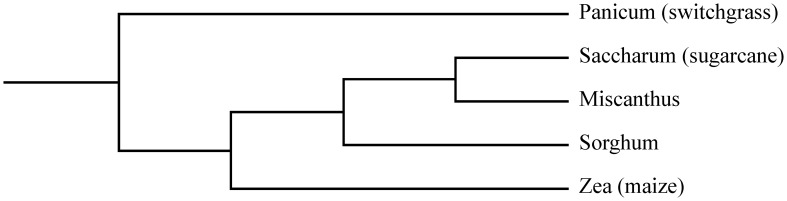
**Phylogenetic tree depicting the relationships between the C4 grasses maize, miscanthus, sorghum, sugarcane and switchgrass.** Adopted from Lawrence and Walbot (2007).

Many of the plant species that generate high yields of biomass with minimal inputs are C4 grasses. C4 plants dominate hot, open, arid environments around the world. The vegetation in these environments consists mainly of grasses and thus it is not surprising that about half of the world's grass species use C4 photosynthesis (Sage et al., 1999). Economically important food crops such as maize (*Zea mays* L. ssp. *mays*) and sugarcane (*Saccharum spp*.) are C4 grasses (Figure 1). These crops are important sources of biomass with well-established production chains that can supply large amounts of agricultural residues. Maize is an annual crop mainly cultivated for its grain or silage as a source of food, feed and in recent decades for the production of first generation bioethanol (Bennetzen, 2009). It is the largest crop worldwide in terms of total acreage (FAOSTAT, 2011). Sugarcane, a large perennial grass that can reach heights of over 5 m, is cultivated primarily for its ability to accumulate sucrose in its stems, which is our predominant source of sugar (Tew and Cobill, 2008). It is the largest crop worldwide in terms of tonnes produced (FAOSTAT, 2011) and is exploited on a large scale in Brazil for sucrose-based bioethanol production (Waclawovsky et al., 2010).

Two of the currently leading dedicated biomass crops—miscanthus (*Miscanthus spp*) and switchgrass (*Panicum virgatum* L.)—are also C4 grasses (Lewandowski et al., 2003b). Both are rhizomatous perennials that typically reach heights of 2–4 m and tend to give high biomass yields annually. Miscanthus is a genus comprising 15 species native to regions of eastern Asia, the Himalayas, the Pacific Islands and Africa (Clayton et al., 2002 onwards). The species are closely related to sugarcane (Figure 1; Heaton et al., 2010). The research on bioenergy crops in Europa has been focused on miscanthus (Lewandowski et al., 2000; Heaton et al., 2008b). Due to its high yield potential, the sterile hybrid *M. × giganteus* is currently the main commercially exploited species of this genus for biomass purposes. Switchgrass is a versatile grass species native to North-America, with two major ecotypes: the lowland and the upland type (Sanderson et al., 1996; Casler and Monti, 2012). Due to its origin and prevalence in this region, the majority of research on biomass cropping systems in the United States has been focused on this crop (Heaton et al., 2008b; Parrish et al., 2012). Sorghum [*Sorghum bicolor* (L.) Moench] is another important C4 grass, as it is the fifth most produced cereal crop worldwide (Saballos, 2008; FAOSTAT, 2011). It is cultivated for its grain, sugar-rich stem juice and/or forage biomass depending on the type of sorghum (grain sorghum, sweet sorghum, or forage sorghum) and is gaining increasing research interest as an annual bioenergy crop (Rooney et al., 2007; Saballos, 2008).

Each of these grasses has its strengths and prospects with respect to their use and development as lignocellulose feedstock. In part this is due to the fact that in order to sustain a large scale biomass supply, a wide range of environments is to be exploited—including marginal soils—and in part this is due to the diverse requirements that are posed to bioenergy cropping systems in terms of biomass quality. Different species are expected to be the best choice of feedstock for biomass production in different environments, as a species' productivity is not constant from site to site and the local climate or soil type may provide an advantage or disadvantage from crop to crop. Hence, the efficient and large-scale production of biomass across diverse environments will require a number of lignocellulosic feedstocks, each with a pallet of cultivars, so that a biomass cropping system can be chosen by growers that is optimally adapted to the production environment and processing methodology.

In the following sections the potential of these important C4 grasses—maize, sugarcane, miscanthus, switchgrass and sorghum—in relation to their use as feedstocks for the generation of cellulosic ethanol is discussed.

## Biomass supply; yield and resource use

The future of cellulosic fuels will be determined by our ability to produce large volumes of inexpensive feedstocks without threatening food security or the environment. The combined supply of lignocellulose from organic residues and bioenergy dedicated cropping systems is envisioned to sustain a renewable supply of cellulosic biofuel and other bio-commodities. For each of the designated C4 grasses their lignocellulose yield potential and resource use is discussed and summarized in Table 1.

**Table 1:**
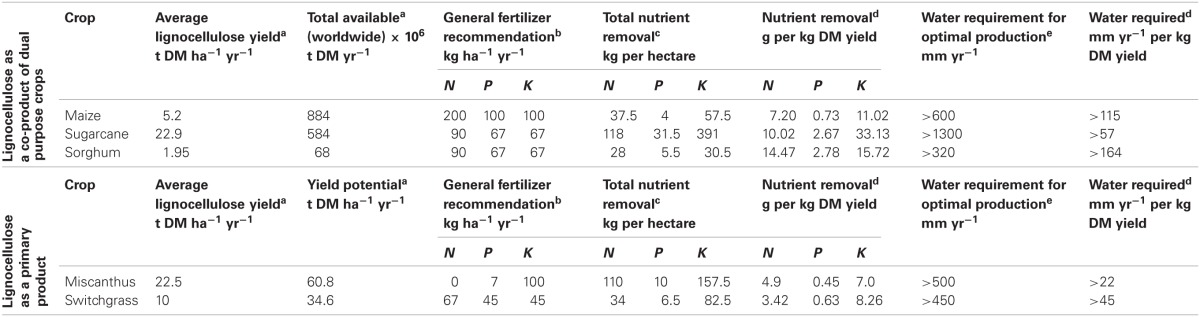
**Average lignocellulose yields and fertilizer and water requirements per hectare and per kg DM yield of important C4 grasses**.

^a^Average yields, total available lignocellulose and yield potentials as reported in sections Lignocellulose as a co-product and Lignocellulose as primary product.

^b^Recommendations for medium fertility soils by University of Georgia's Cooperative Extension Service (Kissel and Sonon, 2008), except recommendation for miscanthus, which is based on Christian et al. (2008).

^c^Data calculated using the average lignocellulose yields per hectare in USDA's Crop Nutrient Tool (http://plants.usda.gov/npk/main, accessed: 20-12-2012), except the values for miscanthus, which are based on Cadoux et al. (2012).

^d^Calculated by taking into account average lignocellulose yield.

^e^Data based on Al-Amoodi et al. (2004) for maize and sugarcane, on Saballos (2008) for sorghum, on Long et al. (2001) for miscanthus and Bouton (2008) for switchgrass.

### Lignocellulose as a co-product

Of the grasses considered here, maize, sorghum, and sugarcane are all contributing to the supply of lignocellulose in the form of agricultural or processing residues. An attempt is made to approach their average residue yield per hectare and their total supply of residue considering their global area harvested.

Maize is primarily cultivated to produce grain, in which case the leaf and stem fractions of the plant (referred to as stover) are available as lignocellulosic residue. A much smaller fraction of maize is cultivated for silage, in which case the whole plant is used and no residue is produced. In the world an area of over 170 million hectares of agricultural land is used for growing maize, with an average grain yield of 5.2 t DM ha^−1^ yr^−1^ (FAOSTAT, 2011). Based on the widely used assumption that the stover to grain ration is 1:1 in maize (Kim and Dale, 2004), the average stover yield is estimated on 5.2 t DM ha^−1^ yr^−1^. Based on these numbers, the potential worldwide biomass supply from maize cultivation adds up to 884 million tonnes. However, the removal of this type of crop residues (normally left in the field) is a delicate issue, as it may increase soil erosion, deplete soil carbon and nutrient reserves and ultimately reduce future crop productivity (Graham et al., 2007). The amount of residue that can safely be removed without jeopardizing soil fertility depends on the cultivation practice (especially tillage regime), and crop yield. With current yield estimates and current rotation and tillage practices in the USA ~30% of the stover may be removed taking into account such considerations (Perlack et al., 2005; Graham et al., 2007).

For sugarcane an estimated total harvested area of almost 25.5 million hectares is reported, with an average yield of 70.5 tonnes ha^−1^ yr^−1^ (fresh cane yield) (FAOSTAT, 2011). Assuming a moisture content of 76% and a bagasse-dry-matter ratio of 0.6:1 as reported by Kim and Dale (2004) the global average bagasse yield per hectare is calculated to be 11.0 t DM yr^−1^. An additional supply of lignocellulose comes from the field residue (leaves, immature stalks and dead tissue), which is estimated to be 65% of the dry stalk yield (Waclawovsky et al., 2010), producing 11.9 t DM ha^−1^ yr^−1^. So even though sugarcane is produced on only one seventh of the land used for growing maize, the total biomass supply from sugarcane cultivation is about two-thirds that of maize, adding up to 584 million tonnes. Taking into account that about 50% of the field residue is commonly left in the field to reduce soil erosion and depletion (Ferreira-Leitão et al., 2010), the available lignocellulosic biomass yield is reduced from 22.9 to 16.95 t DM ha^−1^ yr^−1^.

The global area on which sorghum is cultivated is approximately 35 million hectares, with a global average grain yield of 1.5 t DM ha^−1^ yr^−1^ (FAOSTAT, 2011). With a residue/grain ratio of 1.3 (Kim and Dale, 2004), the stover yield from grain sorghums is on average 1.95 dry tonnes per hectare. The total potentially available supply is thus estimated to be 68 million dry tonnes. However, with the same correction applied to sorghum as to maize, to avoid soil erosion and nutrient depletion, the sustainably harvestable lignocellulose yield of grain sorghum is also ~30% of the stover yield. Another type of sorghum, sweet sorghum, accumulates sugar in its stalks similar to sugar cane and is starting to gain momentum for syrup production (sweet sorghum types) in subtropical regions. These types give fresh yields ranging from 20 to 120 t per hectare (Saballos, 2008). They produce lignocellulose in the form of bagasse and field residue, with average residue yields reported to be 5.8 and 13.9 t DM ha^−1^ yr^−1^, for bagasse and field residue, respectively (Blümmel et al., 2009).

Annually, maize, sugarcane and sorghum can thus globally provide around 1500 million tonnes of lignocellulosic biomass in the form of agricultural residues, from which over 700 million tonnes can be harvested sustainably. Greater productivities are likely with further advances in breeding and production technologies (Perlack et al., 2005). For all these crops, dual-purpose breeding, addressing both grain/sugar and residue yield and quality, will likely take off when the cellulosic ethanol industry adds value to the biomass residue and if these breeding objectives can be advanced simultaneously. Together, agricultural residues from these (and other) crops will make a significant contribution to our global demand for cellulosic feedstocks, and will also play a crucial role in our effective transition toward the production of advanced biofuels (Schubert, 2006; Huber and Dale, 2009).

### Lignocellulose as primary product

Miscanthus and switchgrass are amongst the species with the highest potential as dedicated biomass cropping systems. They are characterized by high dry matter yields and low cultivation inputs and have several advantages as biomass crops due to their perennial nature. There is often considerable variation in biomass yields reported within each species, due to diverse ecological, climatic and cultivation conditions. In addition, side-by-side yield trials with multiple species at various locations and over several years are rare and may fail to assess yields at each species' respective optimum conditions. Therefore we focus on their average biomass yields as reported in literature. However, in order to appraise their yield potential also the most extreme yields observed to our knowledge are reported.

Miscanthus and switchgrass, being herbaceous perennial species, form extensive root systems and have the ability to store nutrient and carbohydrates in rhizomes at the end of the growing season. This supports early shoot emergence and growth in spring (Youngs and Somerville, 2012). Moreover, mature stands of such crops only have to make minor investments into root biomass compared to annual crops. Hence, these grasses, once successfully established, are renowned for their high yield potential (Lewandowski et al., 2003b). In a quantitative review of biomass yields reported for both crops in Europe and the US, miscanthus showed significantly higher biomass yields than switchgrass, with an average of 22 t DM ha^−1^ yr^−1^ from 97 observations, compared to 10 t DM ha^−1^ yr^−1^ from 77 observations, respectively (Heaton et al., 2004).

Only a few trials were set up to assess the yield potential of different miscanthus species, in which *M. × giganteus* often gave the highest autumn yields (Zub and Brancourt-Hulmel, 2010). In a single trial examining the peak yield of *M. × giganteus* under fully irrigated, non-limiting conditions of N, P, and K, a yield of 50 t DM ha^−1^ yr^−1^ was reported in central France (Tayot et al., 1994). A more recent trial in Illinois (USA), surprisingly with minimal agricultural inputs, even reported a peak biomass yield of 60.8 t DM ha^−1^ yr^−1^ (Heaton et al., 2008a), the highest recorded yield of this species to our knowledge. However, in many studies harvest is delayed until winter or even spring and no information on peak biomass yield is collected. Late harvest may reduce yields by on average 33% (Clifton-Brown et al., 2004) and in the worst case by up to 50% compared to peak yields (Lewandowski et al., 2000). Nevertheless, it is a common practice in miscanthus to allow the crops to senesce, in order to let the above-ground biomass dry on the field, and to allow translocation of nutrients to the rhizomes. In general, delayed harvest has a positive influence on biomass quality by reducing water and nutrient content and reduces the removal of nutrients from the system at harvest (Lewandowski et al., 2003a). One of the highest dry matter yields recorded after complete plant senescence was 44.1 t DM ha^−1^ yr^−1^, again in the Illinois field trial of Heaton et al. (2008a).

The maximum yield reported for switchgrass was observed in a United States trial spanning 10 years and several states, in which different switchgrass varieties and harvesting methods were evaluated. In this trial the variety “Alamo” attained a yield of 34.6 t DM ha^−1^ yr^−1^ at a field location in Alabama using a system with two cuts; one harvest around flowering time and another in early spring (McLaughlin and Kszos, 2005). To our knowledge the highest switchgrass peak biomass yield from a single cut trial was recorded to be 26.0 t DM ha^−1^ yr^−1^with the locally adapted variety “Cave-in-Rock” in Illinois (Heaton et al., 2008a). Losses in this species associated with late winter harvest are substantially less than in miscanthus (Heaton et al., 2004).

These yield estimates in all probability represent a baseline, since only limited efforts have been invested in the optimization of crop management and genetics. Although the average yield performance of these species is already impressive, the reported averages are still far less than half of the highest yields reported. This is indicative of the large yield improvements that could be realized in these grasses through breeding efforts, enabling them to advance the production of cellulosic ethanol through the reduction of feedstock costs.

Next to their potential as dual-purpose crops, maize, sorghum, and sugarcane are also envisioned to have potential for the production of lignocellulose as primary product. Specific types are available with reduced grain or sugar yield and increased fiber production, such as forage sorghum hybrids (Venuto and Kindiger, 2008), temperate × tropical maize hybrids (White et al., 2011) and energy canes (Tew and Cobill, 2008), which all have the prospect of high biomass yields. However, at the moment their total lignocellulosic residue supply is the most important driver for the interest in these crops for cellulosic ethanol.

### Inputs of nutrients and water

A high yield potential is a principal requirement for a biomass cropping system. Additionally, those yields are most preferably attained with minimal costs and agricultural inputs, such as fertilizer and irrigation. Moreover, the effects on soil fertility are a relevant issue, since nutrients are inevitably removed from the field at every harvest (Lal, 2005). Nonetheless, the implications of biomass cropping systems on nutrient fluctuations have so far received very limited attention.

Although C4 grasses have an intrinsic advantage over C3 species in terms of water and nutrient use efficiency (Taylor et al., 2010; Byrt et al., 2011; Ghannoum et al., 2011; Sage and Zhu, 2011), considerable differences exist amongst C4 species in their efficiency of biomass production per unit of available resource (Byrt et al., 2011). Crops cultivated for grain production, i.e., maize and grain sorghum, are generally considered to extract more nutrients from the soil, due to the high mineral and protein content of grains (Hons et al., 1986; Shewry and Halford, 2002). Perennials attain higher nutrient use efficiencies than annual crops due to their ability to recycle nutrients to the roots from one growing season to the next (Lewandowski et al., 2003b; Heggenstaller et al., 2009). Consequently, the cultivation of annual crops generally leads to a higher loss of nutrients upon removal of the above ground biomass, as no nutrients are recycled (Byrt et al., 2011). In addition, the extensive root systems of perennials and the reduced tillage compared to annual crop cultivation increase soil carbon content over time, can capture dissolved nitrogen and protect soils against wind erosion (Lewandowski et al., 2003b; Blanco-Canqui, 2010; Dohleman et al., 2010). In the case of miscanthus, nutrients are also returned to the soil through leaf fall prior to the winter harvest (Beale and Long, 1997; Lewandowski et al., 2000). In combination with the early canopy development of established stands, the resulting mulch also aids to prevent the emergence of weeds and reduces the need for herbicides after the establishment phase (Christian and Haase, 2001).

Table 1 provides an overview of recommended fertilization rates and water requirements for the grasses considered in this review. To be able to compare fertilizer/water requirements between these crops it is important to consider the dry matter yield per hectare. To do so, nutrient extraction rates per hectare and minimal annual water requirements are divided by the average dry matter yields reported in sections “Lignocellulose as a Co-Product” and “Lignocellulose as Primary Product.” Note that only lignocellulose yields are considered, making the comparison for maize, grain sorghum and sugarcane somewhat unfair, since fertilizer requirements are developed for grain/sugar plantations. In the table nutrient extraction rates per kg dry matter yield and per hectare are also given, to effectively show the effect of harvesting lignocellulose in each crop on the depletion of nutrients from the soil. These extraction rates are based on the nutrient content of the harvested biomass.

Maize is characterized by an inefficient uptake of nutrients, indicated by the fact that the recovery of applied fertilizer nitrogen is only 37% (Cassman et al., 2002). The replacement of extracted or lost nutrients through fertilization is one of the main production costs in maize cultivation (Berenguer et al., 2009; Subedi and Ma, 2009), although fertilization rates may differ considerably, due to differences in expected yield, local soil conditions and rainfall/irrigation levels (Shapiro et al., 2008).

Sorghum is considered more efficient in its nutrient use than maize, mainly due to its large fibrous root system (Saballos, 2008). It shows only a limited yield response to fertilizer application in medium- to high-fertility soils, and is virtually non-responsive to *P* applications. Hence, fertilization recommendations for sorghum are lower than for maize (Saballos, 2008). Grain and sweet sorghum varieties display similar quantities of nutrient removal as maize, but produce 25–50% higher biomass yields (Slaton et al., 2004; Propheter and Staggenborg, 2010; Propheter et al., 2010).

The high cane yields of sugarcane are often associated with substantial fertilizer applications (Wiedenfeld, 2000; Thorburn et al., 2011). Recommended applications of *N* can be as high as 300 kg *N* ha^−1^ (Roy et al., 2006). Nutrient removal varies considerably, due to environmental differences, different cultivation practices, and large yield differences.

In a comparative study on the nitrogen dynamics in switchgrass and miscanthus, no significant difference between the two species was found (Heaton et al., 2009). In both species mineral contents were high during the growing season, but decreased to minimal levels during plant senescence (Heaton et al., 2009). In a study simulating the impact of a change from unmanaged grassland to switchgrass, it was found that the content of soil organic carbon increased only when adequate *N* fertilizer was applied (Chamberlain et al., 2011). In most soils *P* and *K* levels are adequate for switchgrass (Sanderson et al., 2012). The fertilizer requirements of miscanthus are still under debate (Cadoux et al., 2012). In several experiments miscanthus was shown to give a very limited or no response to *N* fertilizer applications (Danalatos et al., 2007; Christian et al., 2008; Cadoux et al., 2012). Hence, for example, Christian et al. (2008) recommend no *N* application at all. This does not hold true for *P* and *K*, for which recommended applications are 7 and 100 kg ha^−1^, respectively. Davis et al. (2010) hypothesized that miscanthus is capable of nitrogen fixation, explaining the lack of response to *N* applications in these studies. In a review on the nutrient requirements of miscanthus, Cadoux et al. (2012) reported no need for *N* fertilization during the establishment phase of the crop, when yields are expected to be low, but recommend fertilization rates based on typical nutrient extraction levels.

Maize is shown to be the most demanding crop in terms of fertilizer demands, whereas switchgrass and miscanthus are shown to have the lowest requirements for fertilization (Table 1). For grain sorghum and sugarcane similar fertilizer applications are recommended, although sugarcane produces much higher average yields. In addition to fertilization rates, an important parameter is the quantity of nutrients removed from the field at harvest, as this will have an adverse effect on soil fertility levels in the long term if these nutrients are not replaced. It can be deduced from the table that total nutrient (N, P, and K) removal by sorghum and sugarcane are the highest in weight per kg biomass. The lowest quantities of nutrients removed from the field per kg crop are reported for miscanthus and switchgrass. From these figures it can also be deduced that there are large discrepancies in the data between recommended fertilization rates and nutrient removal per hectare, especially for sugarcane. If fertilization recommendations are higher, this may be the result of inefficient uptake of nutrients and/or leaching.

The large-scale production of biomass for biofuel may also have considerable implications on available water resources (Stone et al., 2010). Dedicated bioenergy cropping systems, therefore most likely will have limited possibilities for irrigation and have to rely on rainfall and soil water availability to sustain crop productivity. Therefore, a high WUE is considered a key trait of biomass crops. In general WUE is defined as the dry matter production/loss of soil water (g/kg). However, water loss is not only due to transpiration, but also due to non-biological factors such as soil evaporation. Unfortunately, only a few long-term studies were carried out in these crops that took all these factors into consideration. A further difficulty with the comparison of different studies is the need to normalize findings for differences in the vapor pressure deficit between the inner and outer leaf space (Beale et al., 1999; Jørgensen and Schelde, 2001).

Maize, with a WUE (dimensionless) of 0.0027 is reported to be less efficient than sorghum and miscanthus, with estimates of 0.0038 and 0.0075, respectively (Beale et al., 1999; Long et al., 2001). In another study, miscanthus and switchgrass were reported to have a similar and slightly higher WUE than maize (VanLoocke et al., 2012). The WUE of sugarcane and maize expressed as kg DM ha^−1^ mm^−1^ evapotranspiration where reported to be 17–33 and 7–21, respectively (Berndes, 2002).

Even though WUE may be high in miscanthus and switchgrass, these crops still utilize large quantities of water for optimal crop production. In order to achieve yields of 30 t ha^−1^ with miscanthus, over 500 mm water is required (Long et al., 2001) and even though it is relatively tolerant to drought, miscanthus shows a strong yield response to irrigation at sites with insufficient soil water availability (Price et al., 2004; Cosentino et al., 2007). For switchgrass, economically feasible production is reported to be confined to regions with at least 450 mm annual rainfall (Bouton, 2008). Maize requires roughly 600 mm for optimal production and sugarcane as much as 1300–1600 mm (Al-Amoodi et al., 2004). Sorghum appears to be the least demanding crop, with a water requirement of 320–400 mm (Saballos, 2008).

To compare water use between these crops in a similar way as above for nutrient use, biomass yields have to be taken into account. A crop may require more water, but produce much higher yields than another crop. Therefore, water requirements are reported taking the average yields into consideration and expressed as water requirement per kg DM yield (Table 1). These calculations show considerable differences between the crops, with grain sorghum and maize displaying the highest water requirements per kg lignocellulose produced. Of course, a large part of the water is used in these crops for the grain part of the plant. Amongst the perennial species, miscanthus is shown to produce the highest lignocellulose yield per unit of water.

## Biomass quality

Next to addressing the issues related to the supply of lignocellulose, another important consideration is the efficiency of converting biomass into bioenergy. The challenge of effectively fractionating lignocellulosic feedstocks into fermentable sugars lies within the compositional nature of the plant cell wall. The cell walls of grasses have distinct differences in the balance between the main cell wall constituents (Table 2), even though all commelinoid monocots, including the C4 grasses discussed in this review, share some distinct features in cell wall architecture, as described comprehensively by Carpita (1996), Cosgrove (2005), and Vogel (2008). In each species vast intra-specific genetic variation exists in cell wall composition, polymeric ultra-structure, physical architecture and (presumably) the weight ratio of primary to secondary cell walls. The extent of inter- and intra-specific variation found in these species ultimately indicates opportunities for the development of feedstocks with cell wall characteristics better suited to the demands of the cellulosic ethanol industry.

**Table 2:** **Variation in cell wall compositions of promising C4 energy grasses**^**a**^.

**Lignocellulose feedstock**	**Cellulose**	**Hemicellulose**	**Lignin**	**References**
Maize (stover)	~27–40%	~25–34%	~9–15%	Lorenz et al., 2009b; Templeton et al., 2009; Wolfrum et al., 2009; Lorenzana et al., 2010; Jung and Bernardo, 2012
Switchgrass	~28–37%	~25–34%	~9–13%	Sladden et al., 1991; Vogel et al., 2011
Sorghum (stover)	~21–45%	~11–28%	~9–20%	Rooney et al., 2007; Murray et al., 2008; Shiringani et al., 2010; Stefaniak et al., 2012
Sugarcane (bagasse)	~35–45%	~25–32%	~16–25%	Canilha et al., 2011; Masarin et al., 2011
Miscanthus	~28–49%	~24–32%	~15–28%	Hodgson et al., 2010; Zhang et al., 2012

a Cell wall polymeric values are expressed as a weight percentage of dry matter.

From an economic perspective, feedstocks with the highest combined content of cellulose and hemicellulose (holocellulose) are likely to be favored by the industry, since techno-economic evaluations, and comparative studies of ethanol biorefineries showed that the holocellulose content of feedstocks was directly proportional with ethanol yields under optimal processing conditions (Ruth and Thomas, 2003; Aden and Foust, 2009; Huang et al., 2009). This explains why the predominant strategy in energy grass breeding is to increase the overall abundance of holocellulose in the plant cell wall. As crops like miscanthus, sugarcane and sorghum have in potential a very high holocellulose content on a dry matter basis (~75%), in addition to high biomass yields, they are expected to dominate the future cellulosic ethanol market.

However, current biomass-to-ethanol conversion systems are not optimal and concerns exist as to whether these technologies are universally transferable between different lignocellulosic feedstocks. Leading technologies have almost exclusively been optimized using maize stover and (more recently) switchgrass (Wyman et al., 2005; Elander et al., 2009; Garlock et al., 2011; Tao et al., 2011), with little information available on their applicability in other C4 feedstocks. So far, most efforts to improve biomass-to-ethanol conversion systems have not taken into consideration the impact of biomass composition (Gregg and Saddler, 1996; Kim et al., 2011). Yet, biomass composition may have a large impact on conversion efficiency as, for instance, Kim et al. (2011) demonstrated with compositionally different ecotypes of switchgrass, using the industry's leading pretreatment systems (AFEX, dilute sulphuric acid, liquid hot water, lime, and soaking in aqueous ammonia). Strikingly, the ecotype with the highest cellulose content on a dry matter basis was the worst performer under all test conditions. These results highlight the difficulties of developing universally applicable conversion technologies for different biomass types and indicate the practical limitations of breeding solely for increased levels of cell wall polysaccharides.

Consequently, a second approach to optimize feedstock composition focusses on reducing the natural resistance (biomass recalcitrance) of plant cell walls to enzymatic deconstruction. Significant efforts have been devoted towards understanding and dissecting the biochemical and genetic mechanisms affecting the depolymerization of cell wall polysaccharides. A considerable wealth of studies has documented the extent of natural—and induced—variation of promising C4 grasses with respect to their processing amenability under a diverse array of conversion technologies (Vermerris et al., 2007; Saballos et al., 2008; Dien et al., 2009; Lorenzana et al., 2010; Chuck et al., 2011; Fu et al., 2011a,b; Kim et al., 2011; Lygin et al., 2011; Masarin et al., 2011; Saathoff et al., 2011; Sarath et al., 2011; Xu et al., 2011; Fornalé et al., 2012; Jung and Bernardo, 2012; Jung et al., 2012; Park et al., 2012; Vandenbrink et al., 2012; Yee et al., 2012; Zhang et al., 2012; Torres et al., 2013). Some relevant research highlights are summarized in Table 3.

**Table 3:** **Summary of relevant reports on the variation in conversion efficiency in C4 grass species**.

**Crop**	**Genetic nature**	**Conversion technology**	**Summary of results**	**References**
Maize	Experimental Mapping Population—Hybrid testcrosses of 223 recombinant inbred lines from the IBM collection.	Mild dilute-acid pretreatment followed by hydrolysis with commercial enzyme cocktails.	Variation within population for cell wall glucose release after mild pretreatment and enzymatic saccharification ranged from ~48–56%. Glucose conversion efficiency was strongly correlated to lignin content (*r* = −0.74).	Lorenzana et al., 2010
Sugarcane	Transgenic lines with RNAi-induced down-regulation of caffeic acid O-methyltransferase (*COMT*).	Mild dilute-acid pretreatment followed by hydrolysis with commercial enzyme cocktails.	Maximum reduction in lignin content in transgenic lines compared to controls of 13.7% and a maximum increase in fermentable glucose yield of 35% (after pretreatment and enzymatic hydrolysis).	Jung et al., 2012
Switchgrass	Two sets of genotypes obtained by divergent selection for ruminant digestibility.	Various intensities of dilute-acid pretreatments followed by simultaneous saccharification and fermentation (SSF).	A 40% difference in ethanol yield (after dilute-acid pretreatment followed by SSF) between the two genotypes with the largest contrast in lignin content.	Sarath et al., 2011
Switchgrass	Transgenic lines with RNAi-induced down-regulation of caffeic acid O-methyltransferase (*COMT*).	Various intensities of dilute-acid pretreatments followed by SSF.	Maximum reduction in lignin content in transgenic lines compared to controls of ~15% and a maximum increase in ethanol yield of 38% [after severe pretreatment (0.5% H_2_SO_4_, 180°C) followed by SSF].	Fu et al., 2011a,b
Sorghum	Collection of brown-midrib (*bmr*) mutant collection and their corresponding wild-types.	Mild dilute-acid pretreatment followed by hydrolysis with commercial enzyme cocktails.	Glucose conversion after thermo-chemical processing and enzymatic hydrolysis across a set of 5 *bmr* mutants and their corresponding counterparts ranged from 59–77%. The maximum increase in glucose fermentable yields (relative to wild-type) was of 21%.	Saballos et al., 2008

The majority of studies aiming at the reduction of biomass recalcitrance in C4 grasses has focused on exploring the effect of lignin content on conversion efficiency. Indeed, reductions in cell wall lignin content often led to improved enzymatic digestibility, as shown in studies with *brown midrib* mutants in maize and sorghum (Vermerris et al., 2007; Saballos et al., 2008; Dien et al., 2009; Sattler et al., 2010, 2012; Wu et al., 2011); as well as in studies with transgenes that down-regulate monolignol biosynthesis genes in maize (Park et al., 2012), sugarcane (Jung et al., 2012) and switchgrass (Fu et al., 2011a,b; Saathoff et al., 2011; Yee et al., 2012). In addition to reductions in lignin content, alterations in the ratio between the main constituents of lignin have been found to affect recalcitrance. For instance, a lower S/G ratio—two of the main subunits of lignin—can reduce biomass recalcitrance in C4 grasses, as demonstrated both in natural mutants (Vermerris et al., 2007; Saballos et al., 2008; Sattler et al., 2012) and using transgenic approaches (Fornalé et al., 2012; Jung et al., 2012).

However, lignin content and composition are not the sole factors explaining variation in the conversion efficiency of lignocellulose feedstocks. Several studies on biomass recalcitrance have investigated the impact of differences in the composition and structure of cell wall polysaccharides, and the interactions between polysaccharides and other cell wall components. These demonstrated how cell wall characteristics other than lignin—including the degree of cell wall porosity, cellulose crystallinity, polysaccharide accessible surface area and the protective sheathing of cellulose by hemicellulose—can also contribute to the natural resistance of plant biomass to enzymatic degradation (Mosier et al., 2005; Himmel et al., 2007; Jeoh et al., 2007; Gross and Chu, 2010; Zhang et al., 2012; Zhao et al., 2012).

Furthermore, fundamental cell wall studies in Arabidopsis and other model crops have contributed considerably to the understanding of the synthesis of cellulose and hemicellulose. Consequently, strategies to develop novel genotypes, with reduced recalcitrance, through targeted modifications of cell wall biosynthesis genes are beginning to gain momentum. For instance, alterations in the cellulose synthesis machinery—or its accessory complexes— may lead to modifications in the structure of cellulose microfibrils, with, for example, reduced crystallinity, a lower degree of polymerization and/or a higher degree of porosity. Recently, Vandenbrink et al. (2012) demonstrated a large variation in cellulose crystallinity within a diverse association mapping panel in sorghum, and reaffirmed that genotypes with lower cellulose crystallinity exhibit higher enzymatic hydrolysis rates, as has been reported for pure microcrystalline cellulose samples (Bansal et al., 2010) and ground miscanthus powder (Yoshida et al., 2008). In addition, recent studies that uncovered the function of genes and enzymes in the synthesis and substitution patterns of hemicelluloses provide novel opportunities for the modification of the structural and functional characteristics of hemicellulose (Mortimer et al., 2010; Anders et al., 2012). A possible strategy to improve the processing efficiency of feedstocks aims at a reduction of the number of side-chain substitutions in hemicelluloses, which shield the xylan-backbone from enzymatic hydrolysis (Mortimer et al., 2010).

Despite crucial advances in our understanding of the synthesis and structural properties of the plant cell wall, much still remains to be explored before effective, targeted manipulation of cell wall properties can be fully exploited for the creation of biomass feedstocks optimally suited to bioconversion. Many different pretreatment types exist—and new technologies are developed continuously— that target different components of biomass recalcitrance. Hence, they will require different compositional features of feedstocks for optimal effectiveness. To address this, more research in the area of pretreatment and enzymatic hydrolysis of lignocellulose, as well as research on the intricacies of the cell wall synthesis machinery and on the available genetic variation in cell wall properties within biofuel crops is needed. In particular, quantitative genetic studies and systems biology approaches are anticipated to aid in the understanding of the synergistic and antagonistic interplay of cell wall components and their effect on biomass recalcitrance. The findings from such studies will enable plant breeders to design effective breeding programs and facilitate the development of energy C4 grasses optimized to increase the efficiency of bioconversion technologies.

## Genetic improvement

The C4 grass species discussed in this review are all expected to play an important role as bioenergy crop in the emerging cellulosic ethanol industry. Their success as biomass crop is not only dependent on their biomass yield, efficiency in using resources during cultivation and level of biomass recalcitrance (and other cell wall properties), but also on their amenability for improvements through breeding efforts. In the following sections the improvement of these crops through plant breeding are discussed, with emphasis on crop-specific differences in breeding strategy, selection criteria and tools for breeding and the currently available insights with respect to the genetics of relevant traits.

### Variety concept

Ploidy level and genome architecture are important factors in the design of a breeding program and determine to a large extent the type of variety to be developed. The variety concept therefore is species-specific and apart from commercial considerations it takes into account matters such as mating system, seed production issues, ploidy level and inheritance of traits. Annual crops are generally fertile and are propagated by seeds. If possible, breeding in annual crops aims to generate hybrid varieties to benefit from hybrid vigor (heterosis). Perennial crops are quite often polyploids, with an unbalanced genome constitution causing sterility due to meiotic irregularities. Polyploids tend to be vigorous crops due to a high degree of heterozygosity and gene redundancy.

Polyploidy, however, also complicates genetic studies and the inheritance of traits (Comai, 2005). The mapping and genetic studies of polyploid genomes has to deal with complex levels of allele recombination, especially when chromosome pairing in meiosis is not merely restricted to homologous chromosomes. In addition, as a result of polyploidy sequencing becomes more difficult, due to the large genome sizes and within-genome similarities. Another important consideration is a crop's mode of reproduction, which is a key determinant to which breeding systems can effectively be used to improve a species (Allard, 1960).

Both cultivated maize and cultivated sorghum are diploid species (2*n* = 2*x* = 20) with a basic chromosome number of 10, although other ploidy levels exist in annual and perennial wild relatives in the genus sorghum (Acquaah, 2012b). Maize is predominantly a cross-pollinator (95%) with male and female inflorescences. The former produces pollen which are dispersed by wind (Acquaah, 2012a). Self-pollinations, however, can be done by hand for breeding and research purposes (De Leon and Coors, 2008). The inflorescence of sorghum, in contrast, has separate male and female organs and self-pollination is the main mode of reproduction, with degrees of outcrossing ranging from 5 to 30% (Saballos, 2008).

The perennial grasses discussed here are all wind-pollinated outcrossing species and are characterized by more complex genetics (Vogel and Pedersen, 1993). Switchgrass is highly self-incompatible and possesses a chromosome number of 9, but with varying somatic chromosome numbers and ploidy levels (2*n* = 2*x* = 18 to 2*n* = 12*x* = 108). Amongst lowland ecotypes tetraploids predominate, whereas amongst upland ecotypes octoploids are more abundant (Bouton, 2008). In miscanthus, ploidy levels vary amongst species in the genus, with the three species with the highest potential for biomass production, *M. × giganteus* being a triploid (2*n* = 3*x* = 57), *M. sinensis* a diploid (2*n* = 2*x* = 38) and *M. sacchariflorus* a tetraploid (2*n* = 4*x* = 76) (Heaton et al., 2010). Recently, Kim et al. (2012) reported *M. sacchariflorus* accession from Japan to be typically tetraploid, whereas accessions from China were reported to be typically diploid. *M. × giganteus* is a sterile hybrid, but the other two species are obligate outcrossers due to self-incompatibility (Heaton et al., 2010). All three species are characterized by a basic chromosome number of 19 (Clifton-Brown et al., 2008). Sugarcane is predominantly cross-pollinating, but selfing is possible by covering the inflorescences with bags (OGTR, 2008). The genus *Saccharum* displays a large variation in chromosome number and ploidy levels. The three most important species in the genus used to make modern cultivars are *S. officinarum* (2*n* = 70–140), *S. spontaneum* (2*n* = 36–128) and *S. robustum* (2*n* = 60–200) (D'Hont, 2005; Scortecci et al., 2012). D'Hont (2005) identified a basic chromosome number of 10 for *S. officinarum* and *S. robustum* and a basic chromosome number of 8 for *S. spontaneum*. The genetics of sugarcane and its trait inheritance are very complex, since it is a hybrid of different species and displays both autopolyploid and allopolyploid types of inheritance (OGTR, 2008).

The predicted genome sizes of the C4 grasses vary widely, the smallest being sorghum (1.21 pg), followed by switchgrass (1.88 pg) and maize (2.73 pg) (Bennett and Leitch, 2010). For *S. officinarum* and *S. spontaneum*, genome sizes are predicted to be 3.37 pg and 4.71 pg, respectively (Bennett and Leitch, 2010). The genome size estimations of *M. × giganteus* and its two progenitors species, *M. sacchariflorus* and *M. sinensis* are 7.0 pg, 4.5 pg and 5.5 pg, respectively (Rayburn et al., 2009).

### Genetic resources and breeding tools

There are many differences in the experience, resources and techniques available for each of the crops, giving certain crops distinct advantages over others. Miscanthus and switchgrass have barely been domesticated (Jakob et al., 2009), whereas maize is arguably the most domesticated of all field crops, unable to survive as a wild plant (Acquaah, 2012a).

Maize and sorghum have several advantages over the other crops with respect to their improvement as lignocellulose feedstocks. The complete genomes of sorghum and maize have been released (Paterson et al., 2009; Schnable et al., 2009), while for some of the other grasses sequencing projects are still in progress, such as for *M. sinensis* and switchgrass by the U.S. DOE Joint Genome Institute (JGI, www.jgi.doe.gov/genome-projects). In addition there is a wealth of genomic tools available, especially in maize (genetic markers, genome annotations, quantitative trait loci (QTL's), extensive expressed sequence tag (EST) libraries, well-mapped populations, large collections of mutants) that can be used to study and enhance biomass quality traits. Their diploid nature makes maize and sorghum easier to study than (allo)polyploid crops, and since they are both C4 grasses and have a close evolutionary relationship to the other crops, they are most likely better models to this group of biofuel crops than other model plant species as *Arabidopsis*, rice or *Brachypodium* (Carpita and McCann, 2008). Hence, the knowledge that will be acquired on the synthesis, deposition and recalcitrance of the cell wall in maize or sorghum can most likely be utilized to improve biomass quality of the other C4 grasses. Sorghum shares a high level of co-linearity with the genomes of miscanthus (Kim et al., 2012; Ma et al., 2012; Swaminathan et al., 2012) and sugarcane (Wang et al., 2010), which makes the sorghum genome ideal as a template for comparative genomic studies with these species. In addition, the use of comparative genetics coupled with transcriptomic and proteomic analyses will be an important tool to expedite the genome assembly of closely related C4 grasses. Transcriptome datasets are valuable sources of information to monitor gene expression during different growth stages and biotic or abiotic stress responses. Such datasets can also compensate for the lack of genome sequence information, in those grasses in which sequence information is still unavailable. For example, a large sugarcane EST database is publicly accessible (sucest-fun.org) (Vettore et al., 2003). The combination of genome sequencing with other “omics” strategies is still in its early stages in C4 grasses, but is expected to be a successful strategy for studying cell wall biosynthesis.

Maize and sorghum are the two crops discussed in this review that are annual species, which is likely to positively affect the speed with which these crops can be advanced in breeding programs. Genetic improvement is generally faster in annual crops than in perennials, due to the relatively shorter selection-cycle. All grasses discussed here, including sorghum, can be propagated via outcrossing. This has the advantage that they are amenable to heterosis breeding, in particular when the production of inbred lines is possible by repeated selfings as in maize. An improvement of this technique, nowadays frequently used in maize breeding, is the development of doubled-haploid lines, which are completely homozygous as a result of artificial or spontaneous chromosome doubling of induced haploids (Maluszynski, 2003; Tang et al., 2006). This is a major advantage for hybrid breeding and genetic studies (Forster and Thomas, 2010).

The availability of genetically diverse and advanced germplasm is key to the success of breeding programs. Breeding efforts to improve bioenergy crops can initially take advantage of the knowledge and technologies developed in food and forage breeding programs (Jakob et al., 2009). Maize, sorghum and sugarcane breeding programs have a long history and although these programs mainly target the increase of grain/sugar yield and harvest index, improvements in traits such as disease and lodging resistance affecting yield stability are also useful for their use as dual-purpose crops (Jakob et al., 2009). In forage breeding programs, such as are established in maize, switchgrass and sorghum, the main aim is to improve the total yield of biomass as well as its digestibility. Due to the similarities between enzymatic deconstruction of lignocellulosic biomass in the rumen of cattle and in cellulosic ethanol platforms, crops optimized for forage quality parameters may prove extremely valuable germplasm sources for optimizing biomass quality (Weimer et al., 2005; Dhugga, 2007; Anderson and Akin, 2008; Dien et al., 2009; Lorenz et al., 2009a; Anderson et al., 2010; Sarath et al., 2011).

To expedite the genetic improvement of C4 grass species as lignocellulosic feedstocks, molecular breeding technologies are being considered (Jakob et al., 2009; Takahashi and Takamizo, 2012). Genetic engineering with the help of transformation technologies continues to be a topic of debate, especially in Europe, but public acceptance of genetically modified (GM) crops for dedicated biofuel purposes might be higher than for food and feed commodities. However, transformation technologies are relatively much more developed in dicots than in monocots. Thus for most of these grasses, the exception being maize, major progress is required in the development and optimization of transformation protocols. Reviews on the status of transformation of sorghum (Howe et al., 2006; Girijashankar and Swathisree, 2009), switchgrass (Somleva et al., 2002; Conger, 2003; Bouton, 2007; Burris et al., 2009; Xi et al., 2009; Saathoff et al., 2011), miscanthus (Wang et al., 2011; Engler and Jakob, 2013) and sugarcane (Santosa et al., 2004; Hotta et al., 2010) provide further information. However, transgenic approaches are regarded with great caution in dedicated bioenergy crops as well, as they are mostly outcrossing perennial grasses (Wang and Brummer, 2012). To address the risk of unwanted transmission of transgenes through pollen-mediated gene flow, there are, however, various strategies for gene confinement in perennial biofuel feedstocks (Kausch et al., 2009).

While considerable differences are described between the designated C4 grasses that may affect their improvement as lignocellulose feedstocks, the fact that these crops are evolutionary closely related provides great opportunities for the exchange of acquired knowledge between them. Several online services have been developed to facilitate this exchange of information, e.g., GRASSIUS, a platform integrating information on transcription factors and their target genes in grasses (www.grassius.org) (Yilmaz et al., 2009), GRAMENE, a comparative genome mapping database for grasses (www.gramene.org) (Ware et al., 2002; Liang et al., 2008) and CSGRqtl, a comparative quantitative trait locus database for Saccharinae grasses (http://helos.pgml.uga.edu/qtl/) (Zhang et al., 2013). Hence, advances in each of these crops may expedite research progress in the other crops, with maize and sorghum being anticipated to serve as models in the study of cell wall recalcitrance.

## Prospects and research needs

The group of C4 grasses regarded in this paper has a great potential for the sustainable, large scale production of lignocellulose to support a cellulosic fuel industry. The supply of biomass from different sources and niches will prove to be indispensable, as different growing conditions and refinery technologies require different types of lignocellulose feedstocks. These grasses are likely to represent different sources of biomass supply as they have distinct prospects and potential roles in the future supply chain of lignocellulose, which stipulates the importance of research into the genomics, genetics, and breeding of this group of promising grasses.

Globally, the cultivation of maize, sugarcane and grain sorghum can sustainably provide around 1500 million tonnes of lignocellulosic agricultural residues per year. Greater yields are likely with advances in breeding and production technologies (Perlack et al., 2005), especially when the lignocellulose fraction becomes an important product and dual-purpose breeding sets off. Common plant breeding research needs in such crops for advancing the use of agricultural residues for cellulosic ethanol production focus on (1) increasing the yield of harvestable biomass without jeopardizing food/feed production, (2) exploring the effect of the use of crop residues on soil quality and (3) improving the biomass quality of the residue for bioprocessing. To make the conversion of agricultural residues economically attractive, it is critical that advances are made in biomass quality, in addition to technological improvements in the refinery processes (see also Box 1). Maize and sorghum are the crops that will most likely serve as models in the research on biomass quality improvement, due the presence of the required expertise, genetic resources, proper breeding tools and the availability of their genome sequences. Together, agricultural crop residues can make a significant contribution to our global supply of lignocellulose for biofuel production (Schubert, 2006; Huber and Dale, 2009).

Box 1 **Added-value products in biorefining.**

A key factor that will most likely influence the economics of the cellulosic ethanol industry is the production of different bio-commodities in addition to ethanol, utilizing the diversity of compounds present in biomass. Several high-value chemicals can be produced, some of which may in fact provide greater economic returns than ethanol. However, the value of such commodities is determined to a large extent by market-demand and their value may be reduced when the industry grows to a larger scale. To our knowledge, no research has been conducted to compare different C4 grasses for the production of such bio-products. Reviews on the different products and production routes can be found in—amongst others—(Gallezot, 2007; Fitzpatrick et al., 2010; Deutschmann and Dekker, 2012).

As the industry matures, dedicated energy crops are needed that can be cultivated with limited agricultural resources and grown on surplus cropland and on degraded or marginal soils. Under these provisions, fast-growing perennial C4 grasses have been coined as the most promising candidates for the industrial production of lignocellulosic biomass (Hill, 2007; Carroll and Somerville, 2009). Switchgrass and miscanthus are commercially attractive because of their high biomass yields, broad geographic adaptation, climatic hardiness, efficient nutrient use and nitrogen fixation capacities (Sanderson et al., 1996, 2006; Hill, 2007; Yuan et al., 2008; Tilman et al., 2009; Heaton et al., 2010). Since their cultivation is expected to require low mineral-nutrient inputs and pesticides, these crops are also expected to have high net energy gains and major environmental benefits. As breeding in these crops is still in its infancy, there is most likely ample room for improvement.

The success of C4 grasses in the cellulosic ethanol industry will rely on the production of superior cultivars that increase the profitability and competitiveness of the industry while sustainably meeting projected market volumes. Common breeding objectives, regardless of species or cropping system, include increasing stem biomass yields and cell wall polysaccharide content, as well as reducing the recalcitrance of biomass to industrial processing. Cellulosic grasses, particularly those destined to marginal soils, will be required to combine improved resource use efficiency (water and nutrients), broad climatic adaptation and biotic-stress hardiness.

Although, the above mentioned targets are universal, the advances in breeding programs are different for each species and the initial research focus will be species-specific to ensure an important role for each of the C4 grasses in the future cellulosic ethanol industry.

### The case of maize

As the largest crop worldwide in terms of total acreage (FAOSTAT, 2011), maize is expected to play an essential role in the development and wide-scale commercialization of cellulosic fuels (Schubert, 2006; Vermerris, 2009). This requires the breeding of maize as a dual-purpose crop, displaying optimal grain yield and quality characteristics, as well as high stem-biomass yield and improved processing amenability. Lewis et al. (2010) demonstrated that grain yield, agronomic fitness and stover quality were not mutually antagonistic breeding targets, and concluded that current maize breeding programs could incorporate stover traits interesting to the cellulosic ethanol industry without having to resort to exotic germplasm. With a wealth of agronomic and genomic resources, the possibilities of advancing maize as a dual crop with desirable biomass quality characteristics and a high stover yield are plentiful (Carpita and McCann, 2008). Due to the availability of such resources, its use as a forage crop and its widespread cultivation, producing tons of lignocellulosic residues, maize is most likely the best model crop in the research on biomass quality. The primary research goal in maize bioenergy research lies thus in the dissection and understanding of biomass recalcitrance and the targeted manipulation of cell wall composition. In addition, recent research endeavors are also investigating the potential of maize as a dedicated biomass crop with the development of temperate × tropical maize varieties that produce much higher biomass yields, much lower grain yield and accumulate sugar in the stems (White et al., 2011; Dweikat et al., 2012).

### The case of sorghum

Sorghum is a unique species, in which both grain-types, sugar-types and biomass-types exist (Rooney et al., 2007; Saballos, 2008; Serna-Saldívar et al., 2012). Together with the availability of its genome sequence, this opens up opportunities for sorghum to become a model crop for research on the production of both first- and second-generation biofuels (Olson et al., 2012). The highest lignocellulose yield potential in sorghum exists in forage sorghums (Vermerris and Saballos, 2012). They may provide a good alternative to perennial cropping systems, as they can provide similar dry matter yields, while offering the advantage of an annual growth cycle with respect to the choice of new planting material and the possibility to make changes in the crop rotation system in use.

Sweet sorghums types are also of interest, in particular in areas where sugarcane is already being produced, as the same equipment and processing facilities can be used. They may provide several advantages over sugarcane in terms of resource use efficiency, abiotic stress tolerance and due to its annual nature and simpler genetics.

Enhancing sorghum as a bioenergy crop can be accomplished through a combination of genetics, agronomic practice and processing technology. A particular research objective in sorghum is to increase the germination of seeds at low temperatures, and the ability of seedlings to withstand low temperatures; these cold-tolerance traits will enable earlier planting and therefore extend the growing season, potentially giving rise to higher biomass yields.

### The case of miscanthus

Miscanthus has a high potential for biomass production over a wide range of climates. However, the triploid hybrid *Miscanthus* × *giganteus* is currently the only commercially grown species in the genus. This hybrid, a vegetatively propagated clone, is sterile and lacks genetic variation. It is crucial to broaden the genetic base of the germplasm to be able to extend its geographical adaptation and advance miscanthus for bioconversion and biomaterial applications and as a precaution against potential future infestation with insect pests (Clifton-Brown et al., 2008; Heaton et al., 2010). In addition, being reliant on vegetative propagation, either through tissue culture or through rhizome division, the generation and handling of the planting material of this sterile clone leads to high establishment costs (Christian et al., 2005).

To broaden the genetic variation, attempts are made to resynthesize this interspecific hybrid by making new crosses between its parental species and by searching for more natural hybrids. However, breeding goals are difficult to meet, if the end products of the breeding process are sterile. A way out of this impasse is a focus of the breeding efforts on the development of seed-propagated varieties in genetically stable and fertile species, such as *M. sinensis*. This is economically attractive, as this will most likely lower the costs of planting material considerably, result in a better establishment and speed up the development of miscanthus as a dedicated biomass crop. The self-incompatibility system in miscanthus allows breeders to fix heterosis in the form of hybrid varieties. Alternatively, the creation of hexapoid *M. × giganteus* may also provide opportunities for the production of fertile germplasm (Yu et al., 2009).

### The case of sugarcane

Sugarcane is one of the most efficient crops in the collecting solar energy and converting it into chemical energy (Tew and Cobill, 2008) and as the largest crop worldwide in terms of tonnes produced (FAOSTAT, 2011), its potential as a biomass feedstock is widely acknowledged. In sugarcane breeding efforts have focused on increasing the yield of stem juice volume and stem juice sugar content. However, as stem yield is positively correlated to stem juice yield, lignocellulose yield has to some extent been indirectly advanced (Singels et al., 2005). Current breeding efforts take several directions: breeding solely for sugar content, breeding for its use as a dual crop (energy cane type I) and breeding solely for biomass yield (energy cane type II) (Tew and Cobill, 2008). These energy canes are being generated possessing a higher percentage of alleles from the high fiber, low sucrose species *S. spontaneum* (Ming et al., 2006). Moreover, Inman-Bamber et al. (2011) disproved that these types can attain high biomass yields because of a low sucrose content, rejecting the widespread hypothesized feedback inhibition of sucrose content on the efficiency of photo assimilation. Hence, increases in biomass yield through breeding and selection doesn't necessarily come at the expense of sucrose content. Another challenge in sugarcane breeding is its envisioned geographic expansion to more temperate environments. Advances in sugarcane genetics are challenged by its large autopolyploid genome, organized into variable numbers of chromosomes. Hence, the construction of genetic maps and the identification of molecular markers for the targeted traits will play an important role to improve selection of sugarcane varieties and to speed up the breeding process.

### The case of switchgrass

Switchgrass is adapted to a wide range of climates and efforts to enhance switchgrass for bioenergy purposes benefit from a history of forage breeding (Mitchell et al., 2008). Since switchgrass currently falls behind most of the other crops in terms of lignocellulose yields (section Lignocellulose as primary product), productivity is the single most important objective in switchgrass. Due to the major investments in switchgrass research in the USA and due to the extensive variation present in the species, there is a lot of potential for improvement in this versatile crop.

Significant heritable variation has been shown to exist in biomass yield and related traits (Taliaferro, 2002; McLaughlin et al., 2006; Boe and Lee, 2007). Yield improvements may be achieved in a number of ways. Tiller density and mass per phytomer were shown to have large direct effects on biomass yields in a path analysis, and may have potential as indirect selection criteria for enhancing biomass production in switchgrass (Boe and Beck, 2008). There is a large potential for yield increase through heterosis in upland × lowland crosses, producing hybrid cultivars with up to 40% yield increase compared to the parental lines (Mitchell et al., 2008; Vogel et al., 2010).

As switchgrass seedlings grow slowly in comparison to locally adapted C3 weeds (Parrish and Fike, 2005), earlier emergence and seedling vigor are also deemed important traits to the success of switchgrass as bioenergy crop. Issues with seed dormancy and germination are partly alleviated with seed treatment methods, such as cold storage for 24 months (Haynes et al., 1997), but still remain targets for improvement. Most research is focused on seed size, quality and seedling growth, to enable more successful establishment (Boe, 2003; McLaughlin and Kszos, 2005; Bouton, 2008; Vogel et al., 2010).

## Final remarks

The exploitation of organic residues for the production of cellulosic ethanol may finally become a commercially viable technology, now that research efforts are increasingly devoted to the understanding and improvement of biomass quality. Equally significant is the progress that has been made in the identification and development of dedicated lignocellulose feedstocks. However, we are still far from the ideal of high yielding, resource efficient and stress-tolerant crops that can be sustainably cultivated in diverse environments and produces lignocellulose with a favorable balance of carbohydrates and a low level of recalcitrance. It is important to stress here that it is highly unlikely that a single crop will be able to attend this wide variety of agronomical and physiological requirements. The C4 grasses discussed in this review are envisioned to be the key players in the future supply of lignocellulose, due to their productivity under diverse ecological conditions and because they include both dual-purpose and biomass dedicated crops. Still, their evolutionary relationship and common characteristics may open ways to speed up research progress, for instance through comparative genomics and the exchange of acquired knowledge and resources. As a group C4 grasses are amongst the most promising plants for biofuel production, containing highly productive, resource-use efficient species, harboring great genetic diversity. Maize, miscanthus, sorghum, sugarcane, and switchgrass will all play a central role in the future biomass supply chain for the production of biofuel and other byproducts, and their improvement as lignocellulose feedstock will contribute to the commercial success of the cellulosic ethanol industry.

### Conflict of interest statement

The authors declare that the research was conducted in the absence of any commercial or financial relationships that could be construed as a potential conflict of interest.
